# Inferring temperature adaptation from thermal performance curves of somatic growth rate: The importance of growth measurements and mortality

**DOI:** 10.1111/jeb.14136

**Published:** 2022-12-09

**Authors:** Sigurd Einum, Vitalija Bartuseviciute, Erlend I. F. Fossen, Christophe Pelabon

**Affiliations:** ^1^ Centre for Biodiversity Dynamics, Department of Biology Norwegian University of Science and Technology Trondheim Norway; ^2^ Department of Biological Sciences University of Bergen Bergen Norway; ^3^ Oslo Centre for Biostatistics and Epidemiology, Institute of Basic Medical Sciences University of Oslo Oslo Norway

**Keywords:** climate, comparative ecology, local adaptation, reaction norms, thermal adaptation

## Abstract

When comparing somatic growth thermal performance curves (TPCs), higher somatic growth across experimental temperatures is often observed for populations originating from colder environments. Such countergradient variation has been suggested to represent *adaptation to seasonality*, or shorter favourable seasons in colder climates. Alternatively, populations from cold climates may outgrow those from warmer climates at low temperature, and vice versa at high temperature, representing *adaptation to temperature*. Using modelling, we show that distinguishing between these two types of adaptation based on TPCs requires knowledge about (i) the relationship between somatic growth rate and population growth rate, which in turn depends on the scale of somatic growth (absolute or proportional), and (ii) the relationship between somatic growth rate and mortality rate in the wild. We illustrate this by quantifying somatic growth rate TPCs for three populations of *Daphnia magna* where population growth scales linearly with proportional somatic growth. For absolute somatic growth, the northern population outperformed the two more southern populations across temperatures, and more so at higher temperatures, consistent with *adaptation to seasonality*. In contrast, for the proportional somatic growth TPCs, and hence population growth rate, TPCs tended to converge towards the highest temperatures. Thus, if the northern population pays an ecological mortality cost of rapid growth in the wild, this may create crossing population growth TPCs consistent with *adaptation to temperature*. Future studies within this field should be more explicit in how they extrapolate from somatic growth in the lab to fitness in the wild.

## Graphical abstract

When comparing somatic growth thermal performance curves (TPCs), higher somatic growth across experimental temperatures is often observed for populations originating from colder environments. Such countergradient variation has been suggested to represent adaptation to seasonality. Using modelling, we show that this conclusion is based on assumptions that may not always be satisfied. We illustrate this by quantifying TPCs for three populations of Daphnia magna where detailed information can be used to predict how somatic growth TPCs may translate into population growth TPCs.

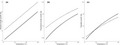

## 1 INTRODUCTION

A commonly applied approach to study how populations have evolved in response to their local temperature regimes (e.g. along latitudinal gradients) is to collect organisms from different populations and measure their somatic growth rates in a common environment at different temperatures to obtain thermal performance curves (TPCs, Angilletta et al., 2003; Khelifa et al., 2019; Montagnes et al., 2022). TPCs may differ in their minimum and maximum temperature allowing positive growth, and the temperature at which growth is maximum (Jonassen et al., 2000; Levinton, 1983). Correlations between these characteristics and temperatures experienced by the populations in the wild are then indicative of local *adaptation to temperature*. However, a perhaps more common result in such studies is that somatic growth (or similar traits such as development time) TPCs of different populations are more parallel and differ primarily in the height of curve (i.e. intercept) (e.g. Campos et al., 2009; Laugen et al., 2003; Schultz et al., 1996; Van Doorslaer & Stoks, 2005; Yamahira & Conover, 2002). Higher growth rate at all temperatures is often observed for populations originating from colder environments. This pattern, known as countergradient variation (Conover & Schultz, 1995), has been suggested to reflect a general selective pressure for a higher growth rate in colder environments, being driven by the requirement to reach a certain size prior to the end of the shorter growth season in these colder climates, and thus represents local *adaptation to seasonality* (Conover & Present, 1990).

Somatic growth rate is an important fitness component because it can increase size at maturation for indeterminate growers with a fixed age at maturation and/or reduce age at maturation for organisms with a flexible age at maturation, two traits that influence birth rate, and in turn population growth rate. However, studies of TPCs have measured somatic growth rate either on absolute scale (e.g. absolute growth in length: [final length–initial length]/time, e.g. Schultz et al., 1996, or absolute growth in mass: [final mass–initial mass]/time, e.g. Yamahira & Conover, 2002), or on proportional scale (proportional growth rate in mass: ln [final mass/initial mass]/time, e.g. Van Doorslaer & Stoks, 2005). Here, we use modelling to show that the choice of scale for expressing growth rate (absolute or proportional) may be crucial for interpreting comparative studies of somatic growth TPCs in terms of adaptation to local temperature regimes (*adaptation to seasonality* vs. *adaptation to temperature*). Furthermore, we illustrate this using an empirical example. Thus, this study highlights an important issue with the interpretation of existing work within this field.

## 2 MODELLING THE FITNESS CONSEQUENCE OF DIFFERENCES IN SOMATIC GROWTH TPCS

In this modelling, we consider studies that measure growth rate in mass and for simplicity we focus on the part of the curve where the increase in somatic growth with temperature is monotonous and can be approximated by a linear relationship on some specific scale. Thus, in this case the TPC is defined by an intercept and a slope. Mass growth can be measured either as a proportional rate (*g* = ln(*M*_*final*_/*M*_*init*_)/*t*) or as an absolute rate (*G* = (*M*_final_ – *M*_init_)/*t*), where *M*_init_ and *M*_final_ are initial and final mass, and *t* is the time interval between the two measures. We assume that for the focal species, population growth rate (i.e. per capita birth rate—mortality rate) scales linearly with proportional mass growth as established for *Daphnia* in a lab study (e.g. Lampert & Trubetskova, 1996). We first consider an experiment where somatic growth rate is expressed on a *proportional scale*, and where thermal performance curves have a common slope (*β*) but different intercepts (*α*_*i*_) among populations (*i*). The TPC in somatic growth can thus be expressed as *g*_*i*_ = *α*_*i*_ + *βT*, where *T* is the temperature. If the relationship between population growth rate and proportional somatic growth rate has a slope of *δ*, the population growth rate of population *i* is given by *r*_*i*_ = *δg*_*i*_ = *δ*(α_*i*_ + *βT*). The derivative of this function with respect to temperature is *dr*_*i*_/*dT* = *βδ*. Thus, the slope of the population growth rate TPC is independent of the intercept (*α*_*i*_) of the proportional somatic growth rate TPC, which means that the effect of temperature on population growth rate is identical among these populations. Thus, for this hypothetical experiment, examination of somatic growth TPCs suggests adaptation to seasonality, and this holds true also when translating these into population growth TPCs.

Next, we consider an experiment in the same species where the somatic growth rate TPCs have a common slope and different intercepts among populations when expressed on an *absolute scale*. To translate these into population growth, we first need to convert absolute somatic growth rates into proportional somatic growth rates, since these latter scale linearly with population growth rates for the species in question. Experiments quantifying thermal performance curves typically standardize starting size among populations and temperatures to remove this source of variation for growth. Absolute growth rate is then linearly related to final size, but proportional growth rate increases asymptotically with increasing absolute growth rate (and final size). This is shown by the derivative of the proportional growth rate with respect to final size which is given by *dg*/*dM*_*final*_ = 1/*M*_*final*_ *t*. Thus, the relationship between proportional and absolute growth rate can then be expressed as *g*_*i*_ = *G*_*i*_^*τ*^, *τ* < 1. If the TPC is based on absolute growth rate, the expression for the population growth rate is then *r*_*i*_ = *δg*_*i*_ = *δG*_*i*_^τ^ = *δ* (α_*i*_ + *βT*)^τ^. The derivative of this function with respect to temperature is *dr*/*dT* = *βτδ* (α_*i*_ + *βT*)^*τ‐1*^. Thus, in this case the slope of the population growth rate TPC depends on the population‐specific intercept of the somatic growth rate TPC (α_*i*_). This means that the effect of temperature on population growth rate differs among populations even if their somatic growth rate responses to temperature are identical. Specifically, given *τ* < 1, populations with a higher elevation of the absolute somatic growth rate TPC will have a shallower population growth rate TPC. This means that even if population differences in absolute somatic growth rates across temperatures are constant, this will translate into differences in population growth rate that diminish with increasing temperature.

**FIGURE 1:**
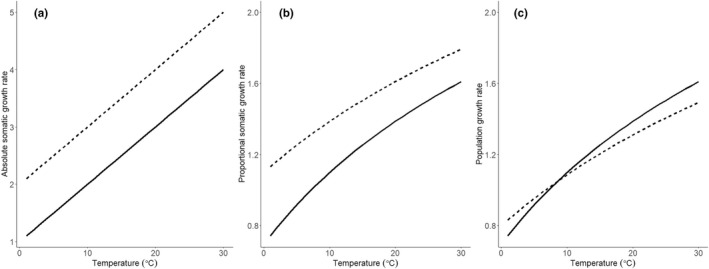
Illustration of correspondence between different types of thermal performance curves (TPCs). In (a), TPCs with respect to absolute somatic growth rate (*G* = (*M*_*final*_‐*M*_*init*_)/*t*, *M*_*final*_ = *M*_*init*_ + *α* + *βT*) differ only in intercept (*α* = 1 and 2 for solid and dashed line, respectively, other parameters common for both populations are *M*_*init*_ = 1, *β* = 0.1, *t* = 1). Traditionally this would be interpreted as evidence for *adaptation to seasonality*. However, when expressed as proportional somatic growth rate (*g* = ln(*M*_*final*_/*M*_*init*_)/*t*)), these TPCs also differ in slope (b). Furthermore, when population growth rate scales linearly with proportional somatic growth rate and the population with higher somatic growth rate pays an ecological mortality cost in the wild, these somatic growth rate TPCs translate into population growth rate curves that may cross (c). Thus, this may be more appropriately explained by *adaptation to temperature*. Population growth rate in (C) is calculated as *r* = *δg* – *m*, where *m* is an ecological mortality cost paid by the fastest growing population (*δ* = 1 and *m* = 0.3 in this example).

So far, we have only considered how temperature influences population growth rate through effects on somatic growth and mortality in a controlled laboratory setting. Although mortality rates observed in the lab can be used to calculate population growth rate TPCs, these will not necessarily reflect the ecological mortality costs of rapid somatic growth that may occur in the wild, and particularly not those that are due to increased exposure to predators when attempting to grow fast (Biro et al., 2004, 2006). Under such a cost, a trade‐off exists between somatic growth and mortality (reviewed by Dmitriew, 2011), such that populations can only evolve a higher somatic growth at the cost of increased mortality. In this case, population growth TPCs may cross within the experimental thermal range, a pattern consistent with *adaptation to temperature*, even when absolute somatic growth TPCs established in the lab only vary in intercept (Figure 1). Thus, for the experiment described above, although examination of absolute somatic growth TPCs suggests *adaptation to seasonality*, population growth TPCs may show evidence for *adaptation to temperature*. This demonstrates the challenge of separating between these two types of adaptation based on somatic growth rate TPCs measured in the lab.

The above derivations show how using measures of somatic growth rate which scale linearly with population growth rates makes it easier to interpret comparisons of somatic growth rate TPCs. We further illustrate this by quantifying somatic growth rate TPCs for three populations of *Daphnia magna*. This study shows that in terms of both absolute and proportional somatic growth rate, a northern population outgrow two more southern ones, suggesting *adaptation to seasonality*. However, if the northern population has evolved a higher somatic growth rate at the cost of increased mortality in the wild due to a trade‐off between these, population growth rate TPCs may become consistent with *adaptation to temperature*.

## 3 METHODS

### 3.1 Study species and populations

*Daphnia magna* Straus 1820, is a small freshwater crustacean that reproduces parthenogenetically under favourable conditions, and that switches to sexual reproduction and production of ephippia containing resting eggs towards the end of the growth season in seasonal environments (i.e. onset of winter or drought). Sediment samples containing *D. magna* ephippia were obtained from three populations: a pond in Værøy, Norway (67.687° N 12.672° E), a pond in Park Midden‐Limburg, Zonhoven, Belgium (50.982° N 5.318° E), and a rice field which is flooded and dries out annually in the Delta del Ebro, Riet Vell, Spain (40.659° N 0.775° E). In the following, these three populations are referred to as the Norway, Belgium and Spain populations, respectively.

### 3.2 Experimental design

We used 10 clones (originating from 10 different ephippia) from each population in the experiments, and these were reared at 17°C with a 16L:8D photoperiod for three to four parthenogenetic generations prior to the experiment. Since these clones result from sexual reproduction they are genetically unique, and can thus be treated as independent biological replicates. During this period, individuals were fed three times a week with Shellfish Diet 1800 (Reed Mariculture Inc.) at final concentration of algae 4 × 10^5^ cells/ml, and the ADaM medium was changed once a week. For the experiment, second or later clutch neonates were collected and photographed less than 24 h after birth. After photographing, neonates were placed individually in 50 ml tubes containing 17°C ADaM medium. Each tube was placed in a Memmert Peltier‐cooled incubator IPP 260plus (Memmert, Germany). We used a 16L:8D photoperiod and the temperature in different cabinets was set to 12.0, 15.0, 17.0, 19.0, 22.0, 24.0 and 26.0°C. Each temperature treatment received eight individuals from each of the 10 clones. Animals were fed every second day with concentrations that had previously been established to represent ad lib rations for the Norway population and that we assumed would also be valid for the other two populations (×10^5^ cells/ml): 12°C, 2.00; 15°C, 2.38; 17°C, 2.62; 19°C, 2.88; 22°C, 3.24; 24°C, 3.50; 26°C, 3.76. The wide range of temperatures used in this study requires temperature‐specific rations to be used. The alternative would be to give a single common ration at all temperatures that ensures ad lib conditions at the high temperature (where requirements are highest), but this would lead to overfeeding at the colder temperatures and deteriorating medium quality that would negatively influence growth.

Due to logistic constraints, the different temperature treatments were run simultaneously for one population at a time (Norway May–June 2015, Spain December–February 2018, Belgium July–September 2018). Although this is unlikely to influence the slopes of the somatic growth TPCs, some variation in their intercept among populations may be caused by a time‐effect. However, one of the clones from the Norway population used in the current experiment (clone 49) was also used by Issa et al. (2022) following the same experimental protocol at a similar temperature (20°C) to one of the temperatures (19°C) used in the current experiment, allowing for a comparison of temporally separated estimated growth rates. These estimates (mean [SD]) were similar, 0.30 [0.03] day^−1^ and 0.33 [0.04] day^−1^ for the current experiment and Issa et al. (2022), respectively, suggesting little potential for such time‐effects on the TPC intercepts in our lab. Although we do not have similar temporally separated data from the other populations, it is unlikely that those would show a different response to time. All individuals were checked daily for mortality and sexual maturity (presence of eggs in the brood chamber). Tubes were rotated daily within the climate cabinets during the maturity checks to avoid positional effects. On maturation, individuals were photographed and terminated.

Gut length (GL, mm, measured from the top of midgut to the hindgut when animal is relaxed) was measured from the photographs for each individual at the neonate and maturity stages using ImageJ v1.48 (National Institutes of Health, Bethesda, MD). Based on this measurement, dry mass (DM, mg) was estimated from GL based on the relationship between these estimated for the Norway population by Fossen et al. (2018): DM = 0.00679*GL* ^2.75^ (*r* ^2^ = 0.99). We assume that this relationship also holds for the other two populations. Proportional somatic growth rate (g) was calculated as *g* = ln (DM_mature_/DM_neonate_)/time, and absolute growth rate (*G*) as *G* = (DM_mature_ – DM_neonate_)/time, where time is the age at maturity in days.

### 3.3 Statistics

Both absolute and proportional somatic growth rates were modelled as a function of population, linear and quadratic effects of temperature, and two‐way interactions between population and the two temperature terms. Clone was included as a random factor for both intercept and slope, the latter with respect to the linear temperature term only. For these analyses, temperature was centeed at 12°C (i.e. the intercept of the model can be interpreted as the growth rate at this temperature). This full model was fitted with maximum likelihood using *lmer*, and all alternative simpler models with respect to the fixed effects were fitted and compared with the Akaike Information Criterion corrected for small sample sizes (AICc). The best models were re‐fitted with restricted maximum likelihood for parameter estimation.

## 4 RESULTS AND DISCUSSION

**FIGURE 2:**
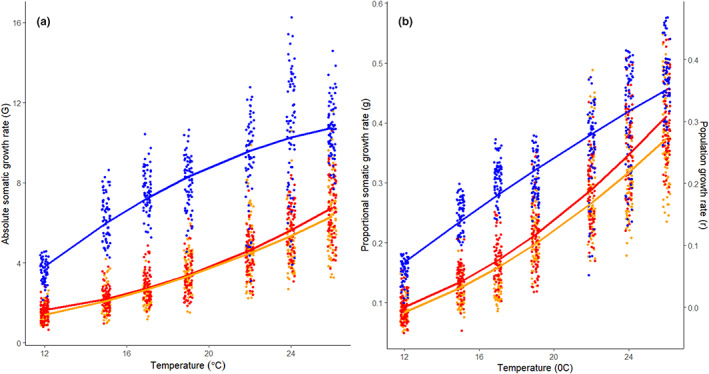
Thermal performance curves of three populations of *Daphnia magna* (blue = Norway, orange = Belgium, red = Spain) with respect to absolute growth rate (μg.d^−1^, a), and proportional growth rate (d^−1^, b). For latter relationship, the secondary y‐axis gives population growth rate based on the linear relationship between proportional somatic growth rate and population growth rate (d^−1^) given by Lampert and Trubetskova (1996) as *r* = 0.967 *g*–0.089. Fitted lines are based on the parameter estimates given in Table 1, data points are jittered (with respect to temperature) observations of individuals.

The somatic growth rate of the Norway population was higher than the two more southern populations across all experimental temperatures, whether this was measured as absolute or proportional somatic growth rate. For both measures of somatic growth, the full model was considerably better than the next best model (i.e. without a population‐by‐temperature interaction, absolute growth ΔAICc = 84.3, proportional growth ΔAICc = 20.2). Thus, both the intercept, the slope and the curvature of the temperature effect differed among populations (Table 1;, Figure 2). However, these two measures provided patterns that differed quantitatively. For absolute somatic growth rate, TPCs diverged with increasing temperature, such that the difference among populations was the largest at the highest temperatures (Figure 2a). In contrast, for proportional somatic growth, and hence population growth rate, TPCs tended to converge towards the highest temperatures (Figure 2b). Thus, assuming that population growth rate is an appropriate measure of fitness (Murray, 1985), the fitness advantage of individuals from the Norway population decreases with increasing temperature.

**TABLE 1:** Parameter estimates for the variation in absolute somatic growth rate (*G*, Figure 2a) and proportional somatic growth rate (*g*, Figure 2b) as a function of temperature (°C) and population

	Absolute somatic growth rate (*G*, μg.d^−1^)		Proportional somatic growth rate (*g*, d^−1^)	
Fixed effects	Estimate	SE	Estimate	SE
Intercept Spain	1.627	0.119	0.0922	0.0050
Intercept Belgium	1.388	0.121	0.0830	0.0051
Intercept Norway	3.803	0.142	0.1674	0.0060
Temperature Spain	0.139	0.043	0.0118	0.0018
Temperature Belgium	0.203	0.043	0.0120	0.0018
Temperature Norway	0.794	0.048	0.0235	0.0020
Temperature^2^ Spain	0.016	0.003	0.0008	0.0001
Temperature^2^ Belgium	0.011	0.003	0.0006	0.0001
Temperature^2^ Norway	−0.021	0.003	−0.0002	0.0001
Random effects (SD)				
Clone	0.072		0.0019	
Temperature|Clone	0.055		0.0023	

*Note*: Parameters are direct estimates (i.e. not contrasts) obtained from the best fitting linear mixed‐effect models (fitted using REML).

Our results are consistent with previous studies showing countergradient variation in somatic growth across latitudes (reviewed by Conover et al., 2009) and suggest that this pattern extends to population growth TPCs for *D. magna*. Assuming that population growth rate is closely related to fitness, this observation is challenging to explain from an evolutionary perspective, as the genotypes present in the Norwegian population appear to be superior to the more southern ones across all tested temperatures. One might suggest that the proposed explanations for similar observations in other taxa also apply to these *Daphnia* populations. For example, in the silversides (*Menidia menidia*), the countergradient variation in somatic growth rate observed among populations along the North American east coast (Conover & Present, 1990) is suggested to result from latitudinal differences in size‐selective winter mortality that increased the fitness benefit of being large following the first growth season at high latitude (Munch et al., 2003; Schultz et al., 1998). This may outweigh any mortality costs that evolution of rapid somatic growth rate may incur. Similar mechanisms favouring the evolution of countergradient variation in somatic growth rates have been suggested for other species (Schultz et al., 1996; Yamahira et al., 2007). Common to these examples is that the somatic growth rate influences body size by the end of the first growth season and prior to entering the winter. In contrast, *Daphnia magna* that inhabits seasonal environments goes through several generations of asexual reproduction during the growth season before entering diapause by producing resting eggs when environmental conditions deteriorate (i.e. during winter or drought). Consequently, in this species, rapid growth should not be selected to increase body size at any given point during the year but should rather be selected via its effect on age at maturity and ultimately on population growth rate. Thus, an alternative explanation is required for countergradient variation observed in organisms like *Daphnia* with multiple generations per growing season. One such explanation could incorporate ecological mortality cost of rapid growth (Biro et al., 2006) that would push the population growth TPC for the Norway population downwards. Depending on the magnitude of such a cost, this may result in crossing TPCs, such that the northern population would have a higher population growth rate at colder temperatures, whereas southern populations would have higher population growth rate at warmer temperatures (as in Figure 1c).

It has been argued (Conover & Present, 1990; Shama et al., 2011; Yamahira et al., 2007) that population differences in somatic growth TPCs can be used directly to distinguish between *adaptation to seasonality* and *adaptation to temperature*. *Adaptation to seasonality* is then believed to be achieved by a higher somatic growth across temperatures for populations inhabiting cold temperature regimes, creating a countergradient pattern. In contrast, *adaptation to temperature* is achieved by increasing growth rate at the temperature that the populations most frequently experience, and by reducing growth rate at less frequently encountered temperatures, generating TPCs with different slopes. However, as we show theoretically (Figure 1), a pattern in somatic growth TPCs consistent with *adaptation to seasonality* may translate to population growth rate TPCs consistent with *adaptation to temperature* if (*i*) the chosen measure of somatic growth rate does not relate to population growth rate in a linear fashion and (*ii*) there is a trade‐off between somatic growth and mortality in the wild. Our empirical analyses illustrates this. When based on absolute somatic growth TPC, the advantage of the Norway population over the two more southern populations increases with increasing temperature, a scenario consistent with *adaptation to seasonality*. Yet, for proportional somatic growth and hence population growth, the situation is reversed, with the difference in population growth rate decreasing with increasing temperature. Adding ecological mortality costs of rapid somatic growth in the wild could then theoretically cause population growth TPCs to cross, a result consistent with *adaptation to temperature*. For *D. magna*, these analyses were made possible due to the existence of an empirical relationship between somatic and population growth rates (Lampert & Trubetskova, 1996). Absence of such data likely limits this approach for other taxa, and even for *Daphnia* we lack data on ecological mortality costs of rapid growth which are required for conclusive evidence. Still, we would like to emphasize that distinguishing between *adaptation to seasonality* vs. *adaptation to temperature* requires at least to express somatic growth TPCs on a scale that can be reasonably assumed to be linearly related to population growth. This issue also applies to comparative studies of developmental rate TPCs (e.g. Laugen et al., 2003), because population growth rate (based on birth rates alone) is highly non‐linearly related to age at maturation (Cole, 1954).

Mitchell and Lampert (2000) quantified among‐population variation in somatic growth TPCs in *D. magna* and concluded that this species shows little evidence for thermal adaptation. All their populations, however, were from a more southern locations (latitude ≤60° N). As discussed by these authors, for such more southern populations there may not be a close relationship between the climatic region of a population and the temperature it experiences and adapt to during the growth season (i.e. when not in diapause) because population at low latitude experiencing warmer climate may not be active during the warmest period of the year. In this case, a lower latitude does not necessarily mean that the population experiences higher temperatures during its growth season. Data on maximum temperatures tolerance in *D. magna* illustrate this point, showing a pronounced difference between populations located in Fennoscandia and continental Europe, weaker and non‐linear trends within continental Europe, and little difference between Belgium and Spain (Seefeldt & Ebert, 2019). This is also the case for our Spain population, which lives in a habitat that dries out during summer and hence is winter active. However, both the Norway and the Belgium populations are from permanent ponds, making it more likely that their thermal selective regimes reflect the latitudinal climatic gradient.

To conclude, we argue that care should be taken when inferring mechanisms of selection from common environment studies that compare somatic growth rate (or development rate) TPCs of populations from different climatic regions. For *D. magna*, somatic growth TPCs showed a clear countergradient pattern between the Norway population and the two populations from continental Europe. Such observations have previously been taken as support for *adaptation to seasonality*. Yet, the possible crossing of population growth TPCs resulting from an ecological mortality cost of rapid growth prevents us from rejecting the hypothesis of *adaptation to temperature* for the current study, where the northern population would be favoured at low temperature, whereas the southern populations would be favoured at high temperature. Thus, we urge future studies within this field to be more explicit in how they deal with the extrapolation from somatic growth to fitness.

## AUTHOR CONTRIBUTIONS

**Sigurd Einum:** Conceptualization (equal); data curation (supporting); formal analysis (lead); methodology (supporting); project administration (lead); supervision (equal); writing – original draft (lead); writing – review and editing (lead). **Vitalija Bartuseviciute:** Conceptualization (supporting); data curation (equal); formal analysis (supporting); investigation (equal); methodology (supporting); writing – original draft (supporting); writing – review and editing (supporting). **Erlend I.F. Fossen:** Conceptualization (equal); data curation (equal); formal analysis (supporting); investigation (equal); methodology (equal); supervision (equal); writing – original draft (supporting); writing – review and editing (supporting). **Christophe Pelabon:** Conceptualization (equal); formal analysis (supporting); methodology (supporting); supervision (equal); writing – original draft (supporting); writing – review and editing (supporting).

## CONFLICT OF INTEREST

The authors declare no conflicts of interest.

### PEER REVIEW

The peer review history for this article is available at https://publons.com/publon/10.1111/jeb.14136.

## Data Availability

Data are available at Dryad https://doi.org/10.5061/dryad.z8w9ghxg1.
